# Drilling accuracy evaluation of a mouldable surgical targeting system for minimally invasive access to anatomic targets in the temporal bone

**DOI:** 10.1007/s00405-023-07925-x

**Published:** 2023-04-03

**Authors:** Lena Geiger, M. Geraldine Zuniga, Thomas Lenarz, Omid Majdani, Thomas S. Rau

**Affiliations:** 1 Department of Otolaryngology and Cluster of Excellence EXC 2177/1 “Hearing4all”, Hannover Medical School, Carl-Neuberg-Str. 1, 30625 Hannover, Germany; 2 Ear Medical Group, San Antonio, TX USA; 3 Tecnologico de Monterrey, Instituto de Otorrinolaringologia, Hospital Zambrano Hellion, TecSalud, San Pedro Garza Garcia, Mexico

**Keywords:** Cochlear implantation, Minimally invasive surgery, Image-guided surgery, Micro-stereotactic frame, Surgical template, Drilling accuracy

## Abstract

**Purpose:**

Minimally invasive cochlear implant surgery using a micro-stereotactic surgical targeting system with on-site moulding of the template aims for a reliable, less experience-dependent access to the inner ear under maximal reduction of trauma to anatomic structures. We present an accuracy evaluation of our system in ex-vivo testing.

**Methods:**

Eleven drilling experiments were performed on four cadaveric temporal bone specimens. The process involved preoperative imaging after affixing the reference frame to the skull, planning of a safe trajectory preserving relevant anatomical structures, customization of the surgical template, execution of the guided drilling and postoperative imaging for determination of the drilling accuracy. Deviation between the drilled and desired trajectories was measured at different depths.

**Results:**

All drilling experiments were successfully performed. Other than purposely sacrificing the chorda tympani in one experiment, no other relevant anatomy, such as facial nerve, chorda tympani, ossicles or external auditory canal were harmed. Deviation between the desired and achieved path was found to be 0.25 ± 0.16 mm at skulls’ surface and 0.51 ± 0.35 mm at the target level. The closest distance of the drilled trajectories’ outer circumference to the facial nerve was 0.44 mm.

**Conclusions:**

We demonstrated the usability for drilling to the middle ear on human cadaveric specimen in a pre-clinical setting. Accuracy proved to be suitable for many applications such as procedures within the field of image-guided neurosurgery. Promising approaches to reach sufficient submillimetre accuracy for CI surgery have been outlined.

## Introduction

Conventional surgery involving a mastoidectomy with a facial recess approach still represents current clinical standard for cochlear implant (CI) surgery [1]. The key issue leading to the high complexity of CI surgery is the anatomic position of the facial recess and its relationship and proximity to the surgical drill when accessing the inner ear. Injuring the facial nerve would lead to palsy of mimic musculature, acoustic hypersensitivity, and reduced lacrimation and salivation while injuring the chorda tympani leads to impaired sense of taste [2]. As the established conventional surgery is safe but time consuming and demands for specially trained surgeons [3] a new surgical technique that is faster, less dependent on the surgeon’s experience level, highly accurate and at least as equally safe is desirable.

According to the shift towards minimally invasive surgery seen throughout all fields of surgery, approaches on minimising invasiveness are also found within the field of cochlea implant surgery. In the academic literature approaches are presented on reaching the cochlea via a single drill tunnel through the mastoid referred to as “percutaneous cochlear implantation” [4], “minimally invasive cochlear implant surgery” (minCIS) [5], or”direct cochlear access” (DCA) [6]. This access can be achieved utilizing image-guided surgery systems [7, 8], customized surgical targeting systems a.k.a. microstereotactic frames [4, 8–14] or robotic systems [6, 15–20]. Recently, research has evolved to the description of first successful implantations in patients via a single drill tunnel through the mastoid [13, 14, 16, 21].

In this study, we evaluate our version of an image-guided surgical targeting system using a mouldable template for minCIS. After its initial description and previous validation in artificial bone [10], its accuracy is now analysed for cadaveric specimen under exploration of additional potential error source or change in error impact. The process involved preoperative imaging with an affixed reference frame, planning of a safe trajectory, customizing the surgical template, performing the drilling along the trajectory and postoperative imaging for accuracy evaluation.

## Materials and methods

### Preparing specimen and image-based trajectory planning

**Fig. 1:**
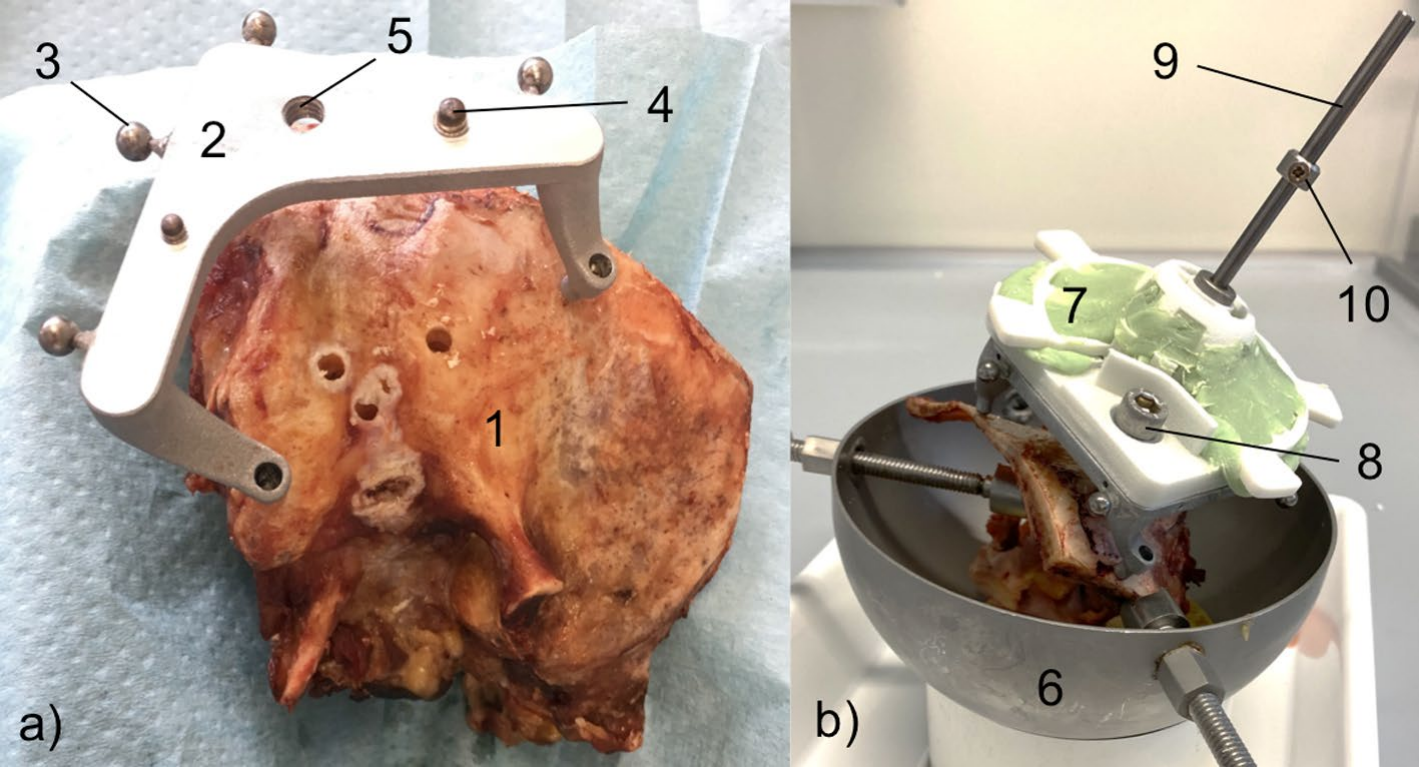
Micro-stereotactic frame. **a** Cadaveric temporal bone specimen (1) with affixed reference frame Trifix (2) and spherical registration markers (3). The dowel pins (4) and a screw hole (5) ensure secure mounting of the template. **b** Specimen with the Trifix is fastened within a mount (6). The mounted template (7) is secured with a screw (8). The step drill (9) is equipped with a set-collar (10) for definition of drilling depth

Four anonymised cadaveric human temporal bone specimens (approval with review board number 3627–2017) were used for eleven drilling experiments. To create a reference coordinate system a reusable reference frame called “Trifix” was mounted on the temporal bone using three bone screws (Max Drive Drill Free 2.0 × 9, KLS Martin Group, Tuttlingen, Germany). The frame was positioned posterior to the outer ear, enclosing the external auditory canal (EAC) (Fig. 1a). Positions of the four spherical registration markers with respect to the dowel pins were determined using a portable coordinate measurement machine (CMM, Romer Absolute Arm Compact 7312, Hexagon Manufacturing Intelligence, Wetzlar, Germany) in order to define a reference coordinate system (CS_3fix_), used throughout the planning and moulding process as well as the evaluation.

For image-based trajectory planning, the specimens were scanned after attaching the Trifix using cone beam computed tomography (CBCT, xCAT, Xoran, Ann Arbor, MI) with 300 μm isotropic voxel size. For better visualisation of the key anatomical structures, in particular the cochlea, chorda tympani and the facial nerve, were manually segmented using MITK Workbench (release 2016.03, German Cancer Research Center, Heidelberg, Germany).

**Fig. 2:**
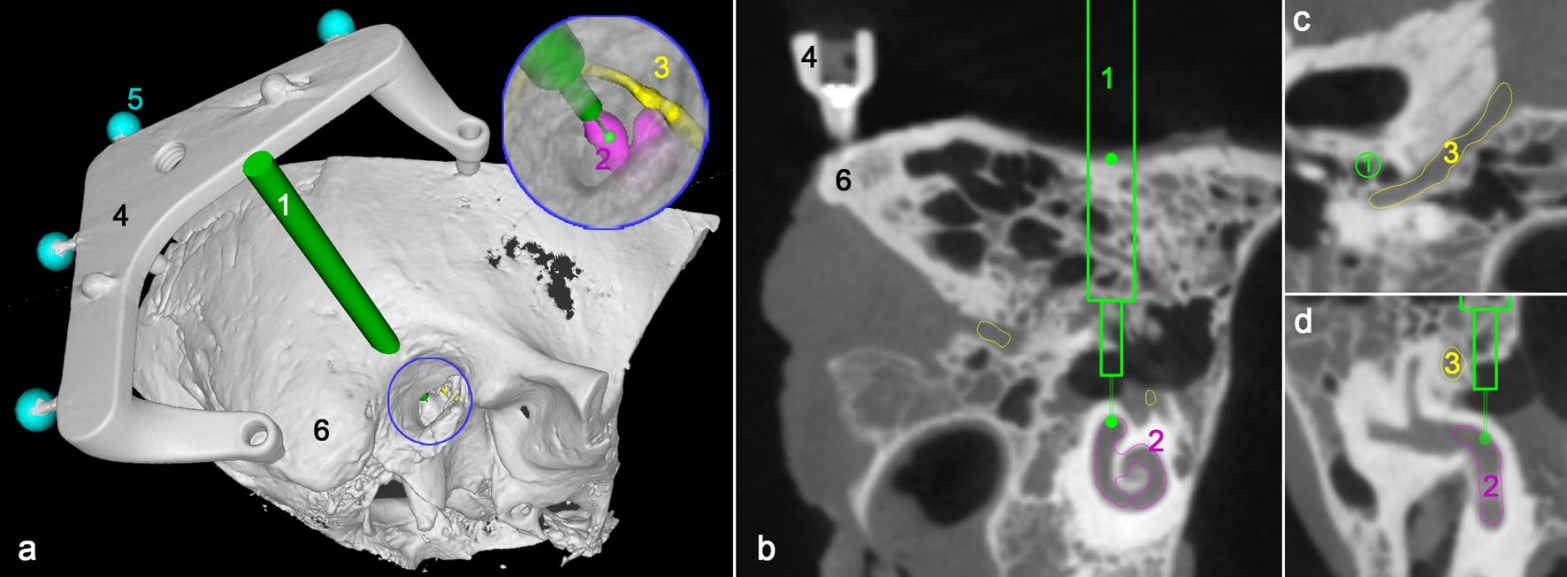
Screenshots of the planning. **a** 3D view of the planned trajectory (1) targeting the cochlea (2) while passing the facial nerve (3). The Trifix (4) with its registration markers (5) is fixed to the temporal bone (6). **b** Sectional view of the CBCT image in the plane of the trajectory, **c** Path of flight view perpendicular to the planned trajectory showing its spatial relation to the facial nerve. **d** Trajectory with planned safety margin to the facial nerve

A planning software developed by our department allows setting visualised trajectories through the temporal bone (Fig. 2). First, a semi-automatic detection of the titanium spheres on the frame was performed and registered to the coordinates (in CS_3fix_) of the CMM measurement done before to enable coordinate transformation. The corresponding registration error was calculated and checked. Next, the trajectory was manually planned with a start point located on the skulls surface and an end point within the temporal bone. For economisation of scarce human specimens more than one experiment was performed on each temporal bone. There is one trajectory leading through the facial recess and a different number of further trajectories per specimen. The different sizes of the temporal bone specimens limited possible positions for Trifix attachment and, therefore, determined the number of total trajectories. Consequently, four of our eleven experiments lead through the facial recess. They are targeting the basal turn of the cochlea and were planned to stop after the facial recess. In terms of planning safety distances to vital structures, highest priority was set on preservation of the facial nerve. For the highest possible comparability, the seven trajectories not leading through the facial recess were planned alongside the recess with a comparable drilling depth. Last, the coordinates of entry and target points of the final trajectory were exported for the subsequent template fabrication process.

### Template fabrication

**Fig. 3:**
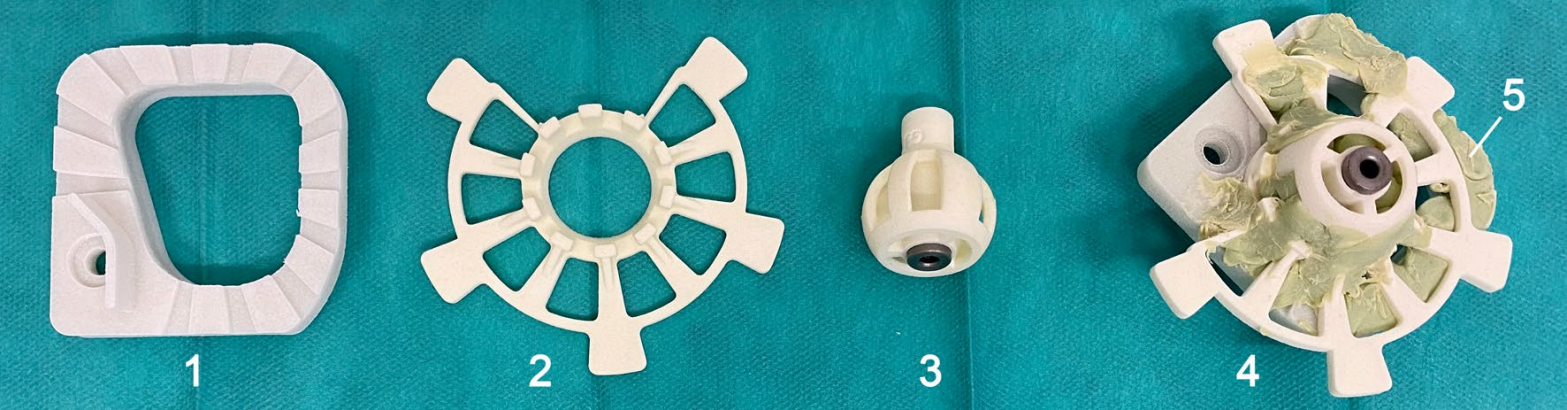
Mouldable surgical template. The base plate (1), subcarrier (2) and drill bushing holder (3) are combined to a patient-specific instrument guide—the “GluingJig” (4). Bone cement (5) guarantees permanent fixation

**Fig. 4:**
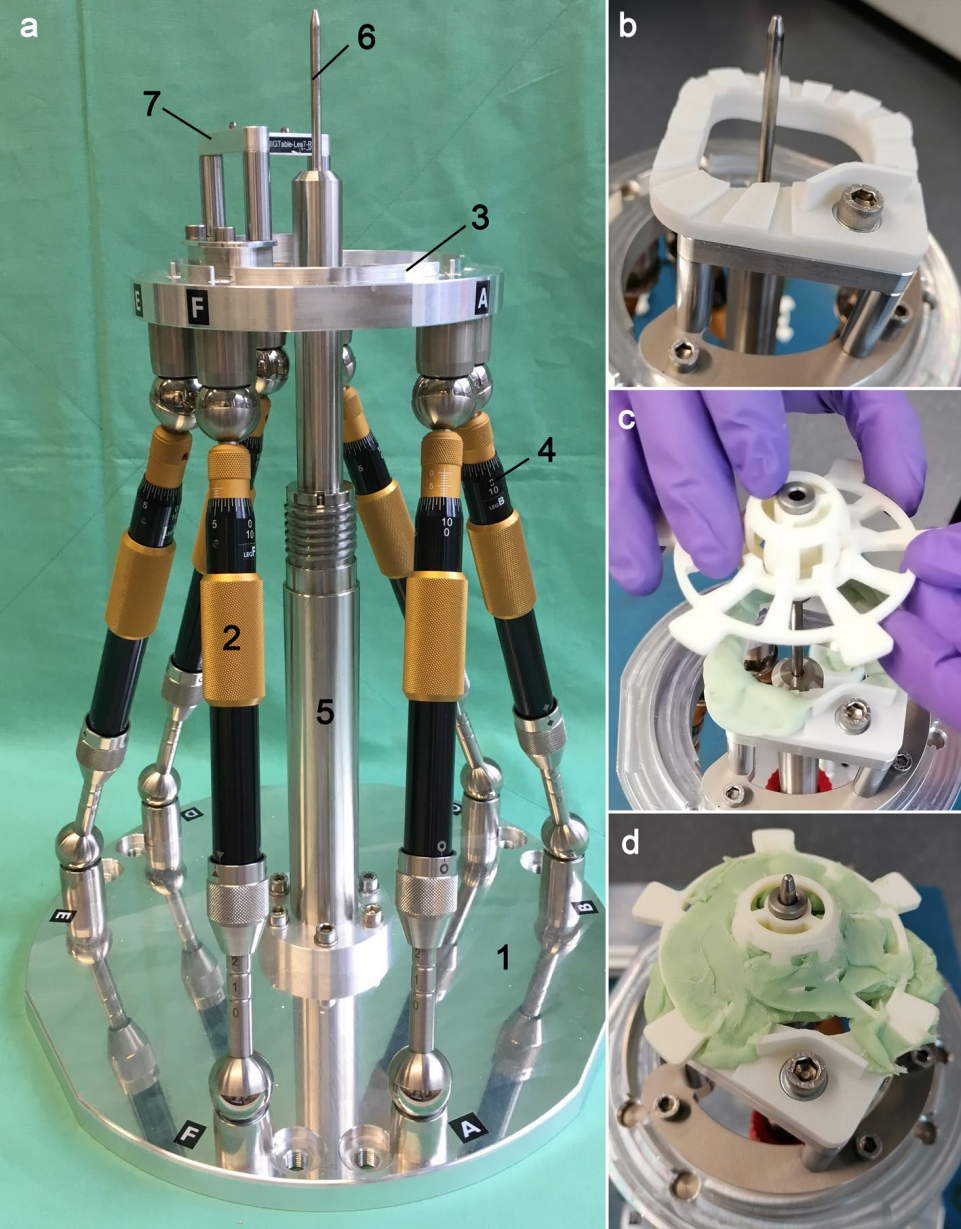
Alignment device and customization of the surgical template. **a** Ground plate (1) is connected by six struts (2) with the moving platform (3). Manually adjustable micrometre screws (4) allow fine-tuning of the length setting. A central pillar (5) ends in the alignment pin (6). It enables patient-specific alignment of the template parts on the mounting table (7). **b** Base plate screwed to the mounting table. **c** Alignment pin ensures patient-specific conglutination of the separate parts. **d** Final surgical template

The mouldable surgical template, called “GluingJig”, consists of three disposable parts (Fig. 3). All were made of polyamide (PA) using selective laser sintering. An alignment device further referred to as “Jig Maker” (Fig. 4), is needed to arrange and temporarily fix the disposable parts according to the individually planned trajectory for permanent fixation with bone cement (Palacos MV, Heraeus Medical GmbH, Wehrheim, Germany). Its construction includes a Gough–Stewart platform with six passive prismatic joints (“struts”) connecting a ground plate with a moving platform. The length of each prismatic joint is manually adjustable resulting in changing position of the moving top platform with the mounting table. The mounting table features the same mounting interface as the Trifix. This allows an accurate transfer of the fabricated jig from the alignment device to the already bone-anchored Trifix. In the centre of the device a pillar with an alignment pin represents the planned trajectory.

Even though all parts of the “Jig Maker” have been fabricated and assembled carefully, manufacturing still introduces inaccuracies and, therefore, deviations from the original computer-aided design (CAD, Inventor Professional, Autodesk, San Rafael, CA, USA) drawings of the Jig Maker. To compensate these inaccuracies in the parametric CAD model of the Jig Maker, which was used for planning of the individual template and calculation of the corresponding length of all six struts of the hexapod, actual dimensions of the assembled device were measured with the CMM. This process is referred to as calibration of the Jig Maker in the following. For manufacturing of the surgical template, the exported coordinates of the planned trajectory were processed using the CAD model providing the specific lengths for the struts. After setting the lengths, the unspecific template parts were placed on top of the mounting table and aligned by the alignment pin in the patient-specific three-dimensional position (Fig. 4b, c). The parts were permanently merged to one surgical template by the above-mentioned bone cement (Fig. 4d).

### Drilling of pre-planned trajectory

When the bone cement’s exothermic reaction subsides (after 15 min on average) the finalised patient-specific template gets mounted onto the patient-affixed Trifix and secured with a screw. Drilling depth was manually adjusted before drilling by equipping the step drill with a set collar (Fig. 1b). The custom-built twist step drills used, showed a narrow part with a diameter of 1.8 mm and a wide part of 4.0 mm. The drill was clamped into a commercial cordless screwdriver (GSR 10,8 V-LI-2, Robert Bosch GmbH, Stuttgart, Germany) and then inserted into the drill bushing of the surgical template, guiding the drill along the planned trajectory. Drilling was performed manually under application of the least thrust possible, to avoid deflecting the drill tip when entering the bone. Leaking bone dust was rinsed with water. Once the set collar reached the top drill bushing and final depth was reached drilling was stopped.

For one planned trajectory the drilling process had to be stopped after passing through the cortical bone, as the drill tip broke. To avoid predictable errors by proceeding drilling into the preformed hole, the experiment was not continued but a new trajectory with the same target but diverging entry was planned instead.

### Evaluation

**Fig. 5:**
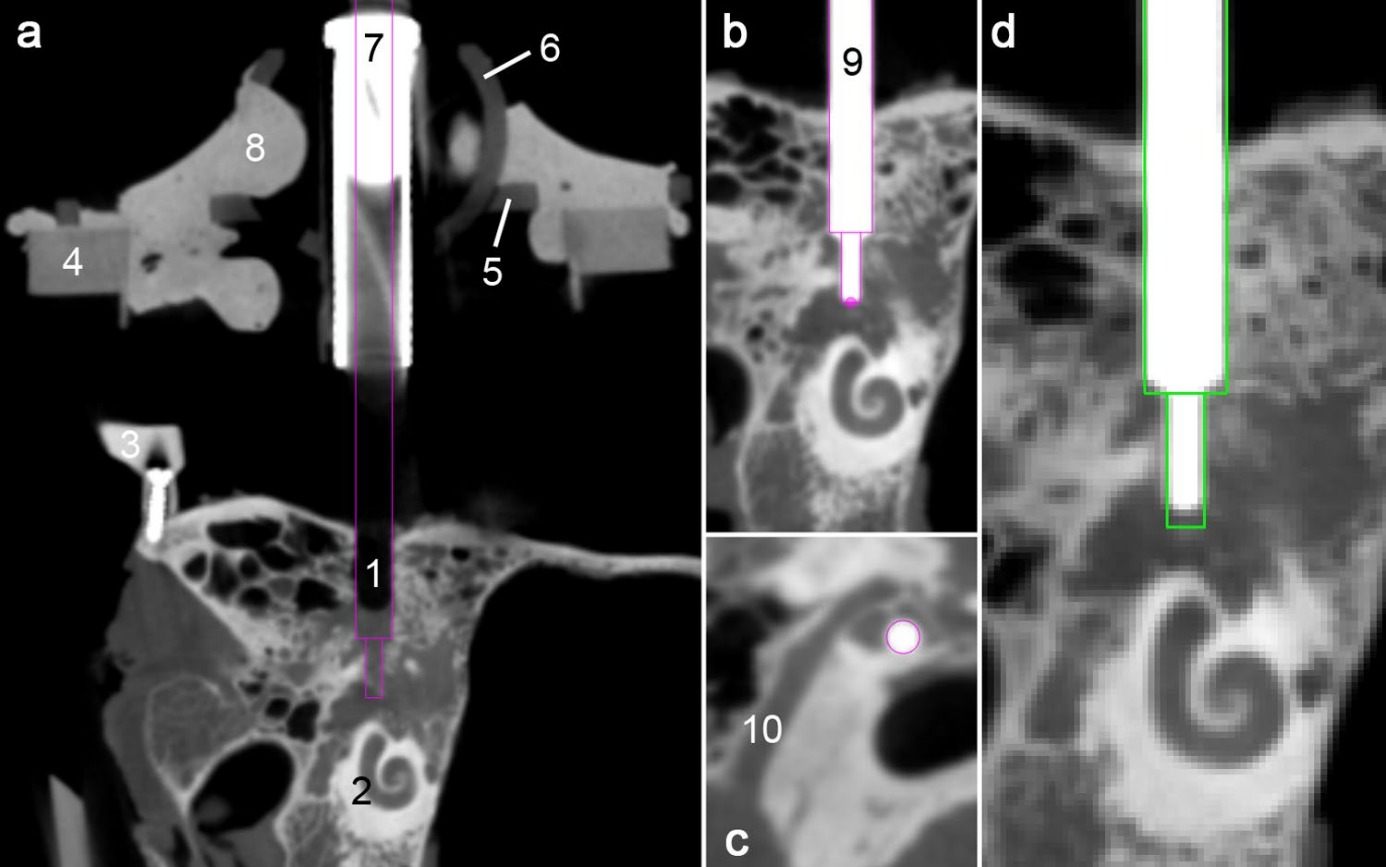
Evaluation of drilling accuracy. **a** Post-experimental image of the drilled trajectory (1) targeting the cochlea (2). The micro-stereotactic frame with the bone-anchored Trifix (3), base plate (4), subcarrier (5), drill bushing holder (6) and drill bushing (7) is also visible. Bone cement (8) infiltrated the cavities inside the separate parts for high stability. **b** Pin inside the borehole used to determine the drilled trajectory. **c** Borehole with sufficient distance the facial nerve (10). **d** Planned trajectory (green) superimposed to the drilling canal

Once the specimen was freed from bone dust a probe pin was inserted into the drill hole. The pin had the same shape as the step drill, therefore, highlighting the drill hole (Fig. 5). After acquisition of a second CBCT scan the software described in the planning process was used for manual determination of the drilled trajectory.

#### Total drilling error

Total drilling error (*ε*_drill_) was determined by comparison of the planned and drilled trajectory utilizing the customized planning software mentioned before. After manual determination of a start and end point in the central axis of the probe pin (Fig. 5) the software issues a trajectory with coordinates relating to the CS_3fix_. In order to reduce inaccuracies from manually marking the pin, its determination was adjusted carefully in several views including the so-called path of flight view (Fig. 5c). After registration of the coordinates of planned and drilled trajectory both trajectories were visualised in the pre scan data set. Deviation between the outer circumferences of both trajectories was measured. Distances between critical anatomic structures and outer circumference of the drilled trajectories were also recorded.

#### Length setting error

To analyse how accurate manual hexapod adjustment was in this series of experiments, lengths for all struts were measured using the CMM after manufacturing the templates and compared to the target values provided during planning. These differences display accuracy and reliability of operating the micrometre screws. This error was calculated as the mean difference of all six struts.

#### Pose setting error

The pose setting error is the deviation between axis of the planned trajectory and actual axis of the alignment pin in relation to the moving platform. The Euclidian distance between both trajectories was calculated in a plane 50 mm below the surface of the Trifix which roughly corresponds to the depth of the cochlea. All error calculations detailed in [10].

## Results

A total of 11 trajectories through the temporal bone have been planned and drilled following the described workflow. Once familiarised with the planning software as well as the hardware, experiments were easily executed by the operator.

In experiment #02 the chorda tympani was injured as planned, given that the narrow anatomy did not allow for preservation of both the facial nerve and chorda tympani. In no other experiments were any other relevant structures injured.

Deviations between drilled and planned trajectories are listed in Table 1. Deviation at the target point was found to be 0.51 mm ± 0.35 mm over all experiments. In addition, for the four trajectories targeting the cochlea, accuracy was also analysed at height of facial recess presenting a mean deviation of 0.55 mm ± 0.38 mm.

**Table 1:** Total drilling error (*ε*_drill_), length setting error and pose setting error

Experiment	Specimen nr	*ε*_drill_ at entry	*ε*_drill_ at target	*ε*_drill_ at facial recess	Length setting error	Pose setting error
#1	1	0.29	0.18		0.01	0.38
#2	1	0.27	0.49	0.44	0.01	0.06
#3	2	0.08	0.23		0.03	0.12
#4	2	0.24	0.73	0.63	0.02	0.05
#5	2	0.28	0.77		0.03	0.01
#6	3	0.63	1.10	1.02	0.01	0.04
#7	3	0.35	1.07		0.01	0.15
#8	4	0.09	0.30		0.02	0.04
#9	4	0.08	0.11	0.11	0.03	0.08
#10	4	0.30	0.37		0.04	0.09
#11	4	0.13	0.28		0.03	0.04
Mean #1–11		0.25	0.51	0.55	0.02	0.09
SD #1–11		0.16	0.35	0.38	0.01	0.10

Minimal distances to anatomic structures to be preserved were assessed for the trajectories passing through the facial recess. Negative results indicate injury to a structure in Table 2. The closest distance of the drill hole to the facial nerve as the most vital structure was 0.44 mm.

**Table 2:** Minimum distances between outer circumferences of drilled trajectory to anatomic structure of interest for trajectories leading through the facial recess

Experiment	Distance to facial nerve	Distance to chorda tympani	Distance to EAC	Distance to ossicles
#2	0.44	− 0.24	1.05	0.39
#4	0.78	0.50	0.63	0.95
#6	1.52	0.67	0.47	1.03
#9	0.76	1.56	0.42	0.46
Mean	0.87	0.62	0.64	0.71
SD	0.46	0.74	0.29	0.33

Negative results indicate injury to a structure

Length setting error was found to be 0.02 ± 0.01 mm. A pose setting error of 0.09 ± 0.10 mm was observed (Table 1).

## Discussion

For minimally invasive approaches at the lateral skull base several concepts have been developed and described utilising image-guidance and robot-assistance within at least the last 15 years [22]. Depending on their level of sophistication, these concepts are tested in different laboratory settings with increasing proximity to intraoperative conditions [23]. Initially, the positioning accuracy is determined in more technical settings [12, 24], followed by drilling experiments in bone-substitute material (“in vitro”, [10, 18, 19]) and in human temporal bone specimens (“ex vivo” [11, 17, 25–27]) before finally reaching the stage of “in vivo” testing of the procedure [4, 28]. Three previously presented approaches [14, 16, 21] have already reached clinical application demonstrating successful implementation of minimally invasive cochlear implantation surgery in patients. Reviews on this topic were recently published by Panara et al. [22] and de Seta et al. [23].

In spite of those promising results, image-guided and/or robot assisted approaches for minCIS are relatively new research topics with several technologies still at an early stage of development [22]—far from having evolved to a well-established clinical standard [11, 23]. Common drawback of all these procedures for minimally invasive approaches on the lateral skull base is that none of them have shown any superior clinical benefit so far. Besides the lack of evidence for improved residual hearing preservation or better speech performance [23] the current state of development even shows inferiority in terms of duration of the surgery [21], unknown complication rates (e.g., risk of facial nerve injury) [22] and an unfavorable cost–benefit ratio [23]. Nevertheless, researchers all over the world are currently driven by the belief that the proposed systems one day will offer higher accuracy, significantly reduced complexity and duration of the procedure finally leading to a higher safety standard of CI surgery—at least for younger and less experienced surgeons.

There are some deficiencies appearing in other concepts for MSF which we want to eliminate or minimise with the proposed concept in the future. We consider a strength of our concept that it enables the feasibility of the minimally invasive approach within a single surgical intervention using intraoperative imaging and intraoperative template fabrication rather than a two-stage surgery with one intervention for attaching bone anchors and a second intervention for the actual temporal bone access [29, 30]. To this end, the proposed concept should ensure template fabrication under sterile conditions as sterilisation processes during surgery costs avoidable extra time. In order to decrease complexity for the surgeons, drilling through the mastoid is guided by a micro-stereotactic frame. Compared to drilling a complete mastoidectomy, setting six micrometre screws, assembling three plastic parts onto the Jig Maker and gluing them together seems to be potentially easier and less error-prone. Since the guided drilling can be performed without visual consideration of the individual anatomy, the single steps of the procedure can be standardized even more, which should also contribute to simplification, saving of time, and increased reliability. In summary, our research is focused on a concept for sterile intraoperative template fabrication demanding high patient safety and user-friendly instrument-guidance in the future.

For cost reasons the separate parts for the mouldable template were made from non-sterile polyamide manufactured by 3D-printing technology. For clinical application they will be designed as sterile disposables. Using injection molding instead of 3D-printing would enable sterile fabrication and a wider choice of materials. Stiffer material in turn could address our assumption that higher rigidity of the base plate might improve accuracy, as deviation under force application seems possible otherwise. The relationships between dimensions of the separate parts, material stiffness and accuracy are subject of ongoing research.

As stated in earlier publications, bone cement is suspected to negatively affect accuracy over time by shrinkage [24]. Two previously formulated corrections to this issue are either reducing the volume of applied bone cement or replacing it by a different adhesive [10]. Another study published later demonstrated a beneficial effect on accuracy after application of super glue instead of bone cement [31]. Nevertheless, there are two opposing hypotheses (H) on how the amount of bone cement might affect accuracy. On one hand (H1), more bone cement should lead to more shrinkage, which reduces accuracy. On the other hand (H2), more bone cement will increase the total stiffness of the template. This reduces vulnerability to lateral forces and could increase accuracy. Since both effects can occur simultaneously with opposing effects on accuracy, it is difficult to make precise statements. In this study, as a precaution, we reduced the amount of bone cement per template and reduced and equalized the time for hardening (15 min ± 3 min). However, the study design does not allow a detailed analysis of the remaining impact of the amount of bone cement on accuracy and further investigation is required, when keeping the use of bone cement and aiming for high accuracy applications.

In comparison with a previous study [10], no remarkable individual errors in manual length setting of the hexapod’s struts have been observed. However, due to imprecise readability of the analogue scale, manual hexapod adjustment stays error-prone despite using the four-eye-principle. A recent study proved an increased accuracy after integrating a digital length measurement system [32]. Integration of such a digital tool could increase safety in the future as it enables detection of user errors.

Since a steady worsening of system accuracy was observed in the experiments up to trial #7, an interim evaluation was performed. Consequently, the Jig Maker was carefully calibrated again prior to trial #8. Furthermore, trials #8 to #11 were executed within 1 day. In contrast, trial #1 to #7 were carried out over several weeks and the Jig Maker was relocated and used for other experiments and student’s training outside this study, which also includes dismounting and reassembling of parts, such as the mounting table and the alignment pin (Fig. 4). Assembly tolerances might explain increasing deviations to the CAD model used for leg length calculation. These potential confounding factors were not considered for the first seven experiments and might explain one aspect of the higher deviation at the target point up to trial #7 of 0.65 ± 0.37 mm, while the accuracy was found to be 0.27 mm ± 0.11 mm for the last four experiments. A learning curve is of course another possible explanation.

Likewise, the interim evaluation could have led to a renewed awareness resulting in an increased carefulness in manual drilling. Drilling deviations also depend on how gentle the drill is advanced and how much thrust is applied. In contrast to robotic devices, this drilling parameter was not technologically controlled in our micro-stereotactic approach and, therefore, could be prone to intra- and inter-individual differences. Using a consumer hand drill with greater weight than an ontological drill might additionally contribute to this effect. Our study can be assumed a worst-case-scenario as increased tool weight and higher manual thrust application worsen rather than improve accuracy. Although we have reviewed all data carefully several times we cannot trace the error back to a single, precisely identifiable cause.

The main purpose of this study was to investigate whether desirable accuracy values can be achieved to access targets in the lateral skull base and to identify error sources to improve systems accuracy in the future. Assessment of suitability depends strongly on the accuracy requirements of the selected procedure. For the originally intended application of minCIS a threshold value of 0.5 mm is commonly used [5, 33]. Based on a population statistics approach by Williamson et al. [34], Schneider et al. [33] concluded sufficient accuracy can be assumed if the systems accuracy (μ + 3σ) is better than this threshold. In this study we failed to present a result underneath this threshold (1.56 mm). However, reducing the accuracy requirements in minCIS to a single numerical threshold does not cover all aspects of anatomical and surgical constrains [5] and thus should be viewed with caution. There are several examples in literature with successful experimental demonstration of a minimally invasive approach with systems not fulfilling this criterion (Table 3). In addition, our system is in an early stage of development and several approaches have been identified to improve accuracy in the future.

**Table 3:** Total drilling error for different of different surgical assistance systems developed for minCIS

Type	Mean	SD	Max	Mean + 3 × SD	n	Material	Year	Study
Robot + IGS	0.78	0.30	1.26	1.68	10	TBS	2009	Majdani et al*.* 2009 [17]
Robot + IGS	0.79	0.56	1.55	2.46	5	TBS	2009	Eilers et al*.* 2009 [15]
Robot + IGS	0.56	0.27	–	1.37	6	ABM	2010	Baron et al*.* 2010 [18]
Robot + IGS	1.40	0.50	2.70	2.90	25	ABM	2010	Stieger et al*.* 2010 [19]
Robot + IGS	0.56	0.41	1.24	1.79	15	TBS	2012	Bell et al*.* 2012 [6]
Robot + IGS	0.15	0.08	0.26	0.39	10	TBS	2013	Bell et al*.* 2013 [26]
Robot + IGS	0.86	0.24	1.29	1.58	8	TBS	2016	Ke et al*.* [20]
Robot + IGS	0.21	0.09	0.34	0.48	9	Patient	2019	Caversaccio et al*.* 2019 [21]
Robot + IGS	0.67	0.27	1.18	1.48	8	TBS	2020	Wang et al*.* 2020 [27]
MSF	0.31	0.10	0.45	0.61	6	TBS	2010	Balachandran et al*.* 2010 [9]
MSF	0.35	0.29	1.08	1.22	10	ABM	2021	Rau et al*.* 2021 [10]
MSF	0.24	0.09	0.36	0.51	10	TBS	2022	Rau et al*.* 2022 [11]
MSF	0.51	0.35	1.10	1.56	11	TBS	2023	present study

*SD* standard deviation, *IGS* image-guided surgery system, *TBS* temporal bone specimen, *ABM* artificial bone material

Regardless of this future evolution of the system, for many other established surgical procedures, such as deep brain stimulation electrode insertion (accuracy requirement of 1.2 ± 0.6 mm to 2.5 ± 1.4 mm) [35] or brain tumor biopsies (accuracy requirement of 1.7 mm) [36] the GluingJig system is already well within accuracy margin. Ball et al. [30], for example, reported the use of a novel stereotactic system for deep brain stimulation. The radial electrode placement error (comparable to our investigated deviation at the target point) was reported to be (0.80 ± 0.41 mm) on average with a maximum of up to 1.66 mm. This range of (in)accuracy was related to “no surgical complications”. Our system is at least as accurate as the device described by Ball et al. [30]; if not even outperforming it.

## Conclusion

We examined the accuracy of our previously presented concept for a mouldable surgical targeting system in human temporal bone specimen. The proposed system has proven its usability for drilling in the irregular mastoid bone. The main concept—patient-specific adhesive bonding of few disposable parts—proved to be suitable not only from a technical but also from a surgical perspective. Sufficient accuracy could be demonstrated for many applications which could motivate a transfer, e.g., to the field of image-guided neurosurgery. For other applications with more demanding accuracy requirements like the originally announced cochlear implantation surgery the system is not suitable in its current implementation. However, promising improvements were herein identified and justify further development.

## Data Availability

The data that support the findings of this study are available from the corresponding author upon reasonable request.
